# Analytical data supporting the “theoretical” postmortem redistribution factor (*F_t_*): a new model to evaluate postmortem redistribution

**DOI:** 10.1080/20961790.2016.1253255

**Published:** 2016-12-16

**Authors:** Iain M. McIntyre

**Affiliations:** Forensic Toxicology Laboratory, Department of the Medical Examiner, San Diego, CA, USA

**Keywords:** Forensic science, forensic pathology, peripheral blood, liver, antemortem, theoretical postmortem redistribution factor (*F_t_*)

## Abstract

The concepts of postmortem redistribution (PMR, *F*) factor, and “theoretical” PMR (*F_t_*) – based upon a drug's characteristic L/P ratio – have been defined to express the direct relationship between postmortem peripheral blood and the corresponding antemortem whole-blood concentration. This paper applies recent data describing liver/peripheral blood (L/P) ratios for many commonly detected drugs to assess these models, and provide a ranking of drugs’ propensity for (and degree of) PMR.

## Introduction

As a consequence of postmortem redistribution (PMR) – due to the movement of the drugs after death [1] – forensic toxicologists have argued a cautious approach in interpreting postmortem blood concentrations [2]. The mechanisms involved in PMR are both complex and poorly understood, but are thought to be explained, to some extent, by the individual physical properties of a drug [3]. When PMR occurs, blood specimens drawn from the central body cavity and heart generally exhibit higher drug concentrations postmortem than specimens drawn from peripheral areas. Diffusion of drugs from organ tissues, muscle and fat into the blood may explain the observed phenomenon [1,4].

In a set of case studies of six drugs, concentrations in the postmortem femoral blood specimens exceeded the antemortem concentrations in five of the drugs studied, suggesting that even peripheral blood exhibited some redistribution [5]. The study did not, however, describe the postmortem interval between death and autopsy. This interval (or postmortem delay) has been proposed to influence PMR [6]. The likelihood for redistribution of other drugs in postmortem peripheral blood has also been documented more recently [7].

In an early attempt to assess and account for PMR, Prouty and Anderson [6] first presented information about blood drug concentrations collected from different sites postmortem. Then, Dalpe-Scott et al. [8] presented a list of drug concentrations from both cardiac and peripheral blood samples expressed as a ratio of cardiac-to-peripheral blood (C/P) for over 100 drugs. The C/P ratio became a benchmark with the accepted guideline that ratios greater than 1.0 were associated with redistribution, and high ratios indicated potential for significant PMR [8,9].

Limitations of the C/P model, however, have been documented. The relationship between C/P and individual drug properties has not been established [10]. In addition, there has been little agreement as to what ratio actually defines a compound as one that is prone to substantial or minimal PMR [11]. Furthermore, reports of a C/P ratio greater than 1.0 have been published for salicylate, carisoprodol, and naproxen, which are not prone to redistribution [5,11,12]. Arterio-venous differences, anatomic variability within individuals, and statistical chance may result in a C/P ratio greater than 1.0 in drugs that do not redistribute. In addition, resuscitation attempts may result in a C/P ratio less than 1.0 [13]. Inaccurate ratios may also be obtained as an artefact of sampling upon depletion of the cardiac blood volume by the collection of blood from connected blood vessels, or in cases of acute overdose where the drug has not undergone complete absorption and/or distribution. Consequently, the traditional C/P ratios can be inconclusive and even misleading with respect to interpretation of PMR [14].

Alternately, the liver-to-peripheral blood (L/P) ratio has been proposed as a more robust marker for PMR. Ratios less than 5 L/kg were presumed to indicate little to no propensity for PMR, and ratios exceeding 20–30 L/kg indicative of a propensity for significant PMR [11]. A number of reports and a literature review elaborating on, and supporting, this model have now been published [14–19]. Furthermore, a direct correlation between the postmortem peripheral blood and corresponding antemortem concentration has been published [20]. Based upon this work, a PMR factor was defined – a factor (*F*) that expressed the direct relationship between postmortem peripheral blood and the corresponding antemortem whole-blood concentration [21]. More recently, this concept was expanded with the development of an equation to determine a “theoretical” postmortem redistribution factor (*F_t_*) based upon a drug's unique L/P ratio – the only independent variable [22].

The current paper uses recently published information of L/P ratios for 44 drugs, and presents additional antemortem and postmortem analytical data, to evaluate and support the derived formula used to estimate *F_t_*.

## Methods

### Autopsy and postmortem specimen collection

In all cases, a full autopsy was conducted at the San Diego County Medical Examiner's Office. Autopsies were performed within 48 hours of the recorded time of death. In cases in which the decedent was found dead, the time of death was recorded as the time found. This was not the exact time of death, which may have occurred several hours earlier. No attempt was made to establish the exact time of death for these cases. However, no cases were included in the analysis if the delay between the exact time of death and the recorded time of death was excessively long (greater than 24 hours). No cases showed obvious signs of decomposition; cases were not included if decomposition was noted by the medical examiner investigator, or observed during the autopsy procedure.

Autopsies on the cases examined in this investigation were performed by board certified forensic pathologists. Although individual pathologists had slightly different approaches to details within the autopsy procedure, the general technique and specimen collection were consistent. The autopsy was started with a usual Y incision to allow viewing of the chest and abdominal organs. Following an initial inspection, each organ was removed for a more detailed examination. During examination of the liver, sections of the right lobe of liver (approximately 100 g) were collected and stored in an opaque plastic four-ounce container without preservative. There was minimal chance of contamination from gastric contents or other sources with this section and collection technique. Upon removal of the intestines, the common iliac vein was visualized and punctured or cut and the peripheral blood specimens (generally 10–20 mL) collected and stored in standard glass tubes containing sodium fluoride (100 mg) and potassium oxalate (20 mg). Using this technique (visual identification of the iliac vein in the pelvis), the pathologist was able to ensure collection of blood returning from the leg. However, as the upper section of iliac vein was not usually clamped, there was potential for a small volume of blood to accumulate from more proximal regions in some cases. Despite this relatively minor exception, there was minimal opportunity for substantial contamination of the blood, especially from other sources. All samples were stored at 4 °C until analyzed.

### Toxicology

In general, the toxicological screening regimen consisted of the analysis of postmortem blood for alcohol and simple volatile compounds (GC-FID headspace), drugs of abuse by ELISA (at a minimum: cocaine metabolite, opiates, methamphetamine, benzodiazepines, cannabinoids, fentanyl) (Immunalysis Inc., CA), and an alkaline drug screen by GC-MS following solid-phase extraction. An acid/neutral drug screen with HPLC-photodiode array detection following specimen precipitation with acetonitrile was performed as required (often dictated by medications found at the scene). Positive results were confirmed and quantified by subsequent and specific techniques. Most of the drugs studied were quantified by an alkaline liquid–liquid extraction followed by GC-NPD detection which has been previously described [18]. Drugs determined by other procedures were amphetamine and methamphetamine determined by GC-MS [23], fentanyl by GC-MS [24], zolpidem and quetiapine which were determined by a high-performance liquid chromatographic method (HPLC) [25], gabapentin by LC-MS [26], and alcohol which was quantitated by GC-FID headspace.

### Deduction of equations

Table 1 presents recently published median L/P ratio data for 867 cases where 44 drugs were examined [19]. Using these data, supplementary investigations were then undertaken to advance the concepts of the PMR factor (*F*) [21], and the “theoretical” PMR factor (*F_t_*) [22].

**Table 1.** Liver/peripheral blood (L/P) ratio data, and theoretical postmortem redistribution factor (*F_t_*): alphabetical listing for 44 drugs.

Drug	*n*	L/P (median)	*F_t_*
Acetaminophen	15	1.1	1.0
Amiodarone	5	41.9	3.6
Amitriptyline	57	16.4	2.3
Amlodipine	9	27.8	2.9
Amphetamine	15	7.0	1.6
Bupropion	11	1.0	1.0
Carisoprodol	11	2.0	1.1
Chlorpheniramine	3	9.5	1.8
Citalopram	37	7.5	1.7
Clomipramine	5	61.0	4.3
Clozapine	12	6.7	1.6
Cyclobenzaprine	13	19.6	2.5
Desipramine	6	44.6	3.7
Dextromethorphan	4	8.6	1.8
Diltiazem	8	17.2	2.4
Diphenhydramine	54	6.7	1.6
Doxepin	20	19.4	2.5
Doxylamine	3	2.9	1.2
Fentanyl	16	5.9	1.5
Fluoxetine	48	30.0	3.1
Gabapentin	28	0.65	0.97
Guaifenesin	5	0.9	1.0
Hydrocodone	38	3.0	1.2
Hydroxyzine	10	12.3	2.0
Imipramine	6	28.8	3.0
Lamotrigine	3	8.5	1.8
Meprobamate	8	0.9	1.0
Methadone	94	4.8	1.4
Methamphetamine	18	6.2	1.6
Metoprolol	6	3.5	1.3
Mirtazapine	5	12.0	2.0
Naproxen	20	1.0	1.0
Olanzapine	20	12.0	2.0
Paroxetine	19	29.2	3.0
Promethazine	9	9.1	1.8
Propoxyphene	35	9.0	1.8
Propranolol	6	10.9	1.9
Quetiapine	65	11.2	2.0
Sertraline	9	76.0	4.8
Tramadol	36	2.3	1.1
Trazodone	19	2.8	1.2
Venlafaxine	42	3.3	1.3
Zolpidem	14	2.4	1.2
Ethanol			1.0

Equation (1) presents the proposed relationship between the antemortem whole-blood concentration of a compound and the corresponding postmortem peripheral blood concentration [21,22]:

(1) $$\text{A}\text{M}=\text{P}/F(or\;F_{t}),$$

where AM is the antemortem whole-blood concentration; P is the postmortem peripheral blood concentration [22];and

(2) $$F_{t}=\sqrt{2(R+2.5)}/2.6,$$

where *R* = L/P ratio (established characteristic drug ratio); L is the liver concentration; P is the postmortem peripheral blood concentration [22].

Using Equation (2), the calculated *F_t_* values for all 44 drugs (including ethanol – from literature data [27]) were established, and are also presented in Table 1. All calculations for *F_t_* were based on the median L/P ratio values [19].

Rearrangement of Equation (1) gives [21,22]

(3) F=P/AM.

Thus, an example of an experimental *F* could be determined for a drug where both the postmortem peripheral blood and antemortem whole-blood drug concentrations have been determined in the same individual (assuming an insignificant delay between the collection of the antemortem blood and the time of death – insignificant opportunity for continued and substantial drug metabolism).

## Results and discussion

**Figure 1.**
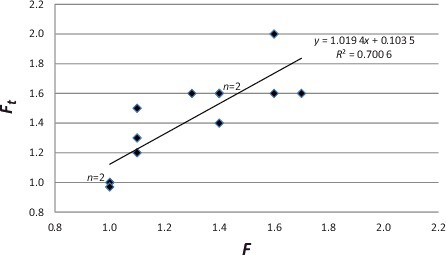
Case examples: postmortem redistribution factor (*F*) compared to theoretical postmortem redistribution factor (*F_t_*).

Table 2 and Figure 1 detail 13 individual examples (nine drugs from nine different cases). A comparison was made between the actual case data (*F* – as determined by comparison of the postmortem peripheral blood “P” and the antemortem “AM” concentrations), and the theoretical (*F_t_* – calculated from Equation (2)). These data showed a statistically significant linear relationship between the determined ratio and the theoretical *F_t_* (*y* = 1.0194*x* + 0.1035; *R*^2^ = 0.700 6; *F*(1,11) = 19.946; *P* = 0.001), thereby providing support for both the concept and the proposed model formula.

**Table 2.** Case examples: postmortem redistribution factor (*F*) compared to theoretical postmortem redistribution factor (*F_t_*).

Drug	P/AM	*F*	*F_t_*	T1	T2
Ethanol	0.28/0.27	1.0	1.0	36	42
	0.059/0.057	1.0	1.0	4	11
Gabapentin	20/20	1.0	0.97	4	11
Fentanyl	0.0016/0.0014	1.1	1.5	11	13
Zolpidem	0.030/0.027	1.1	1.2	17	18
Methadone	0.56/0.39	1.4	1.4	18	6.7
Amphetamine	0.07/0.05	1.4	1.6	7	22
	0.16/0.10	1.6	1.6	9	5.2
Methamphetamine	0.44/0.33	1.3	1.6	7	22
	13.0/9.3	1.4	1.6	7	5.2
	0.34/0.19	1.7	1.6	8	30
Metoprolol	7.0/6.2	1.1	1.3	1	26
Quetiapine	0.24/0.15	1.6	2.0	4	11

P, postmortem peripheral blood concentration (mg/L; ethanol g/dL); AM, antemortem blood concentration (mg/L; ethanol g/dL); *F*, postmortem redistribution factor (actual); *F_t_*, theoretical postmortem factor (using Equation (2)); T1, time between antemortem specimen collection and death (minutes); T2, postmortem delay (hours).

The 13 individual examples examined herein provided essentially optimal circumstances for postmortem toxicological investigation and interpretation. The exact time of death was documented, and there was minimal postmortem delay (time between death and autopsy) – the postmortem delay ranged from 5.2 to 42 hours [average (±S.D.) 17 (±8.4) hours; median 13 hours]. There was no obvious consequence of a change in *F* due to postmortem delay, although the number of cases may be too small to adequately address the issue. Since these postmortem delay times were comparable to those recorded in cases applied to the evaluation of L/P ratios (customarily within 48 hours), data derived from the 867 cases were considered as an appropriate reference source.

Consequently, theoretical *F_t_* values for all 44 drugs (evaluated from the 867 cases) were reproduced. The results were recorded on a continuum of propensity for PMR ranging from the lowest to the greatest (see Table 3). Again, as recognized earlier from observation of the L/P ratio data, drugs previously suspected to exhibit substantial PMR were noticeably differentiated from those thought not to demonstrate such postmortem changes – the higher the *F_t_* value, the greater the propensity for PMR. However, as there is no direct evidence available to confirm that the same relationship exists for the other 35 drugs, this is therefore a speculation in need of additional verification by subsequent investigation(s).

**Table 3.** Theoretical postmortem redistribution factor (*F_t_*): listed in increasing propensity for postmortem redistribution (PMR).

Drug	*F_t_*
Gabapentin	0.97
Ethanol	1.0
Naproxen	1.0
Guaifenesin	1.0
Acetaminophen	1.0
Bupropion	1.0
Meprobamate	1.0
Carisoprodol	1.1
Tramadol	1.1
Hydrocodone	1.2
Trazodone	1.2
Doxylamine	1.2
Zolpidem	1.2
Metoprolol	1.3
Venlafaxine	1.3
Methadone	1.4
Fentanyl	1.5
Amphetamine	1.6
Clozapine	1.6
Diphenhydramine	1.6
Methamphetamine	1.6
Citalopram	1.7
Chlorpheniramine	1.8
Dextromethorphan	1.8
Lamotrigine	1.8
Promethazine	1.8
Propoxyphene	1.8
Propranolol	1.9
Hydroxyzine	2.0
Mirtazapine	2.0
Olanzapine	2.0
Quetiapine	2.0
Amitriptyline	2.3
Diltiazem	2.4
Cyclobenzaprine	2.5
Doxepin	2.5
Amlodipine	2.9
Imipramine	3.0
Paroxetine	3.0
Fluoxetine	3.1
Amiodarone	3.6
Desipramine	3.7
Clomipramine	4.3
Sertraline	4.8

The resulting calculation for *F_t_* (and consequent interpretation of potential for PMR) may not be upheld in all casework, especially on occasion of substantially longer postmortem delay (greater than 48 hours) – particularly for those compounds displaying extensive PMR potential. In such cases, and certainly in the event of decomposition, the possibility of considerable physical and chemical changes may cause additional and inconsistent drug redistribution, thereby increasing interpretative complexity. Furthermore, in cases of overdose, where incomplete distribution of a drug can result in variable concentrations throughout the body's organs and tissues, the current approach may not always be suitable. Such cases, therefore, should be interpreted even more cautiously. Collection procedures employed for postmortem blood and liver tissue samplings are also important matters for consideration. Consistency in the collection technique of postmortem blood is critical. Concentrations of many drugs have been shown to have substantial site-dependence [28]. Concentrations attained from drug analyses performed on heart (or central) blood, pericardial blood, chest blood and perhaps subclavian blood, where drug concentrations can be erroneously elevated, may not be applicable to this particular model and the resulting estimate of the redistribution factor (*F_t_*). Likewise, the site/location of collection of the liver sample is important. Liver concentrations may differ if collected near the lower left quadrant where contamination from gastric content can occur [29]. Nonetheless, if sample collections were consistent and contamination-prevented (or at least marginalized), assumptions and calculations analogous to those made in this paper can be practicable.

Although the approach described in this paper was capable of providing an accurate explanation and interpretation for data collected at this Medical Examiner's Department, it can be anticipated (or even expected) that it will not be possible in all casework to predict a precise antemortem drug concentration. However, a technique to estimate potential for PMR, together with a reference list of some of the most commonly encountered drugs in postmortem forensic toxicology, is now attainable. The resulting classification of drugs’ propensity for – and anticipated degree of – PMR should assist with a rational interpretation of postmortem drug concentrations for forensic experts.
